# Cannabis Influences the Putative Cytokines-Related Pathway of Epilepsy among Egyptian Epileptic Patients

**DOI:** 10.3390/brainsci9120332

**Published:** 2019-11-20

**Authors:** Yasmeen M. Taalab, Wessam Fathi Mohammed, Manar A. Helmy, Alyaa A.A. Othman, Mohamed Darwish, Ibrahim Hassan, Mohammed Abbas

**Affiliations:** 1 Forensic Medicine and Clinical Toxicology Department, Faculty of Medicine, Mansoura University, El-Mansoura 35516, Egypt; dryasmeen.taalab@gmail.com (Y.M.T.); manaradel1982@gmail.com (M.A.H.);; 2 Institute of Forensic and Traffic Medicine, University of Heidelberg, 69115 Heidelberg, Germany; 3 Neurology Department, Faculty of Medicine, Mansoura University, El-Mansoura 35516, Egypt; wesam010@mans.edu.eg (W.F.M.); mohamedabbas017@gmail.com (M.A.); 4 Clinical Pathology Department, Faculty of Medicine, Mansoura University, El-Mansoura 35516, Egypt; mohamad_darwish_79@yahoo.com; 5 Movement and Training Science Department, Institute of Sports Sciences, Johannes Gutenberg University of Mainz, 55128 Mainz, Germany

**Keywords:** cannabis, purified cannabidiol (CBD), tetrahydrocannabinol (THC), epilepsy, inflammatory cytokines, seizures, treatment-resistant epilepsy, anticonvulsant drugs

## Abstract

The study aims to investigate: (1) the prevalence of cannabis among epileptic patients seen at Mansoura University Hospital, (2) serum levels and gene expression of cytokines in epilepsy patients and the controls. and (3) the possibility that cannabis use affects the cytokine levels in epilepsy patients, triggering its future use in treatment. We recruited 440 epilepsy patients and 200 controls matched for age, gender, and ethnicity. Of the epileptic patients, 37.5% demonstrated lifetime cannabis use with a mean duration of 15 ± 73 years. Serum levels of interleukin IL-1α, IL-1β, IL-2, IL-4, IL-6, IL-8, IL-10, and tumor necrosis factor-α (TNF-α), were analyzed and gene expression analysis was conducted only for those cytokines that were different between groups in the serum analysis. The “Epilepsy-only” patients had significantly higher serum and mRNA levels of IL-1α, β, IL-2,6,8, and TNF-α compared to the controls and the “Cannabis+Epilepsy” group (*p* = 0.0001). IL-10 showed significantly lower levels in the “Epilepsy-only” patients compared to the controls and “Cannabis+Epilepsy” (*p* = 0.0001). Cannabis use is prevalent among epilepsy patients. Epilepsy is characterized by a pro-inflammatory state supported by high serum and gene expression levels. Cannabis users demonstrated significantly lower levels of inflammatory cytokines compared to epilepsy non-cannabis users which might contribute to its use in the treatment of resistant epilepsy.

## 1. Introduction

Epilepsy is one of the most common neurological disorders that affect up to 3% of people worldwide and is characterized by the spontaneous recurrence of unprovoked seizures [1,2]. Despite the availability of more than 30 antiepileptic drugs (AEDs), 30% to 40% of patients continue to experience seizures [3], and, consequently, are considered to have pharmacoresistant or treatment-resistant epilepsy (TRE) [4,5]. Furthermore, many patients, whether seizure-free or not, suffer adverse events during the treatment course that are sometimes even worse than the seizures themselves [6]. Therefore, it becomes imperative to find novel therapies that have better efficacy and lesser side effects than the currently available AEDs [7]. In a more general view, current treatment strategies target seizures suppression; however, the underlying pathophysiological mechanisms are not targeted as they are incompletely unraveled. Hence, a better understanding of epileptogenesis may lead to the development of more effective pathophysiology-driven drug treatments [5].

Elevated serum inflammatory mediators have been frequently encountered in many neurological disorders including epilepsy [8,9,10,11,12,13], that gives evidence of the role of inflammation in the etiology of neurological diseases. The involvement of cytokines in the pathogenesis of epilepsy has been proposed by the evidence that limbic seizures increase messenger RNA (mRNA) of inflammatory cytokines in rodent forebrains. In addition, the release of tumor necrosis factor-α (TNF-α) and interleukin-6 (IL-6) from rat hippocampal slices is enhanced by seizures, and an increase in IL-1β immunoreactivity has been found in human epileptic tissue [14]. The crosstalk between inflammation and different epilepsy syndromes considers both the inflammatory state in the epileptic brain as well as increased permeability of the blood–brain barrier (BBB) are the leading cause of enhanced neuronal excitability [15]. Patients with temporal lobe epilepsy and focal cortical dysplasia show elevated pro-inflammatory cytokines levels, suggesting that even in the absence of evident clinical clues, there may be a link between inflammation and epilepsy [16,17,18,19,20,21,22]. Additionally, the use of systemic corticosteroids and adrenocorticotropic hormones as anti-inflammatory immunomodulation therapy has shown successful results in treating specific epilepsy syndromes, supporting the involvement of the immune system in epilepsy [23,24,25,26].

Currently, there has been great interest in the use of cannabis plant extracts as a potential alternative therapy for refractory epilepsy [3,27,28,29,30]. Results of the anecdotal data and early clinical trials suggest improvement in about 50% to 60% of patients who took various cannabis extracts for TRE, including those who were treated with purified cannabidiol (CBD) [31]. Cannabis produces dozens of compounds; termed phytocannabinoids, among which cannabidiol (CBD) is one of the major secondary metabolites lacking the Δ9-tetrahydrocannabinol (Δ9-THC) psychoactive effects [32]. Thus it is considered the main nonpsychotomimetic compound of the Cannabis Sativa plant [33]. Regarding its safety profile, CBD has a low affinity for the cannabinoid receptors (CB1/CB2) and does not act like a typical cannabinoid receptor agonist; thus, it is devoid of the common deleterious effects of Δ9-THC [34,35]. 

Following the characterization of the cannabinoid receptors, CB1 receptors are found predominantly expressed in the brain as well as in the periphery whilst, CB2 receptors are found predominantly in the peripheral tissues of the immune system such as monocytes, B-cells, T-cells, and macrophages [36,37,38], where they are principally mediating the release of cytokines [38,39]. CB2 receptors are also found in the brain, though not quantifiably expressed as CB1 receptors [29]. Interestingly, the CB2 receptor is found primarily on microglia and not on neurons, unlike the CB1 receptor [39]. Afterward, the association between cannabis and the immune system has been widely studied, but it remains unclear whether cannabis inhibits or enhances the immune system and whether or not the relation to the immune system could contribute to seizure regulation/dysregulation. This question is raised since cytokines are the central signaling molecules of the immune system, as well as a primary regulator of inflammation, which is one of the putative mechanisms of epilepsy [40]. 

Therefore, it is not unforeseen that CBD has neuroprotective effects, potent antioxidant and anti-inflammatory activities [41,42,43] exerted by the modulation of a large number of brain and peripheral biological targets (receptors, channels) involved in the development and maintenance of neurodegeneration and hyperexcitability [33]. It inhibits experimental seizures in animal models and improves certain types of intractable epilepsies in patients. However, its pharmacological profile for the treatment of epilepsy is still uncertain [32]. Emphasis on the endocannabinoid mechanisms underlying the neurotherapeutics of CBD in epilepsy is the most common explanation. The therapeutic claims for epileptic seizures are based thus on the probability of CBD and/or the related constituents in the cannabis affecting the endocannabinoid receptors (CB1/CB2) in the CNS and also in the periphery and thereby modulate the inflammatory status and neuronal network involved in the generation and/or spread of hyperexcitability and epileptic seizures [29]. 

Despite the recently published randomized controlled trials (CRTs) of a pharmaceutical formulation of highly purified cannabidiol and its approval by the Food and Drug Administration (FDA) for the treatment of seizures [44,45,46], these regulatory trials are not widely accepted in Egypt. To the best of our knowledge, no studies have been published addressing the Egyptian population. Furthermore, the mechanism by which cannabis could heal epilepsy is yet unclear but could be partially accredited to its anti-inflammatory properties. Therefore, in order to capture a comprehensive profile of the inflammatory pattern in epilepsy, we focused on a wide range of inflammatory markers, including pro-inflammatory cytokines (interleukins IL-1α, IL-1β, IL-2, IL-6, tumor necrosis factor-alpha (TNF-α)), anti-inflammatory cytokines (IL4, IL-10), and chemokines (IL-8). Using an epidemiological comparative study design, we aim to investigate: (1) the prevalence of cannabis among epileptic patients seen at Mansoura University Hospital (MUH), (2) serum levels and gene expression of cytokines in epilepsy patients and the controls; and (3) the possibility that cannabis use affects cytokine levels in epilepsy patients, triggering its future use in treatment.

## 2. Materials and Methods

### 2.1. Participants

The participants were enrolled from the Epilepsy Clinic at Neurology Department-MUH from October 2016 to April 2019. A diagnosis of epilepsy is made by history taking, description of seizure semiology, neurological examination, and electroencephalogram (EEG). Informed consent was obtained from each participant by the treating physician or other members of the research team according to the ethical guidelines. The study was approved by the Institutional Research Board (IRB) at the Faculty of Medicine, Mansoura University, Egypt.

Participants were screened to exclude neurological diseases except for epilepsy, substance use disorders (SUD) except cannabis, psychiatric disorders, cardiovascular illnesses and those who had serious medical illnesses in the past 6 months. Regarding the use of cannabis, the inclusionary criterion includes the regular (cannabis use disorder, CUD) or sporadic/recreational use of cannabis at least 4 times per month for the last 6 months.

### 2.2. Measures

#### 2.2.1. Demographic and Background Information

Demographic information includes age, sex, current/past medical history, cannabis use history and characteristics, and epilepsy status including age at seizure onset, epilepsy duration, seizure type, type of therapy, seizure severity and mean frequency at enrollment, and adverse event profile (AEP) at enrollment.

##### Neurologic Medical Examination

Study questionnaires included the Chalfont seizure severity scale (CSSS) and the AEP [47,48]. All data were collected prospectively using standardized forms and questionnaires. The AEP is a 19-item inventory that assesses anti-seizure drugs (ASD) adverse effects with higher scores indicating more severe adverse events [49]. The CSSS is a measure of seizure severity that assesses the components of seizures most disturbing or disruptive to the patient; it has high interrater and test–retest reliability; a change of 10 points or more on CSSS is considered clinically significant [47].

##### Cannabis Use Survey

All patients seen in the Epilepsy Clinic at Neurology Department-Mansoura University Hospital from October 2016 to April 2019 were asked by the treating physician about cannabis use. Patient who reported the use of cannabis to the physician were screened using cannabis urine testing as per standard clinical care. They were assessed using the drug severity index (DSI) and a timeline follow-back (TLFB) method as an additional method of verifying user status together with a modified nine-item survey on cannabis use to ascertain how patients with epilepsy at a tertiary care clinic in Mansoura/Egypt are using cannabis outside the medical system, and to elaborate on their perception about cannabis use during the course of the disease [50].

#### 2.2.2. Samples and Measurement

Urine samples were collected for drug abuse testing for two purposes—rapid check kits to ensure the inclusion of participants who reported no drug use and confirmatory immunoassay analyses for those who reported cannabis use and showed positive results by rapid check kits. Blood was drawn by trained phlebotomists from all participants for cytokines serum analysis and gene expression analysis. All data were anonymous and were assigned a number, corresponding to the number of the consent form. The results were entered into Excel for storage and subsequent analysis. Patient medical records were completely confidential.

##### Determination of Tetrahydrocannabinol (THC) in Urine Samples

Each urine specimen was added to a mixture of liquid reagents in a DRI® homogenous enzyme immunoassay (EIA) kit (Thermo Fisher scientific). Cannabis metabolites are normally detected in analyzed samples through an immunological interaction with monoclonal antibodies specific for THC.

##### Cytokines Serum Analysis (Inflammatory Biomarkers)

The blood was drawn at 37 °C by venipuncture from EDTA-anticoagulated blood samples. The serum was separated, aliquoted, and stored at −80 °C for subsequent cytokine analysis. The concentrations of the following cytokines were measured using enzyme-linked immunosorbent assay (ELISA) according to manufacturers’ instruction (RayBio Eliza Kits, RayBiotech Life, Georgia, USA): pro-inflammatory cytokines (interleukins IL-1α, IL-1β, IL-2, IL-6, tumor necrosis factor-alpha (TNF-α)), anti-inflammatory cytokines (IL4, IL-10), and chemokines (IL-8).

##### Gene Expression Analysis

Gene expression analyses were only performed for the cytokines found to be different in the serum when compared between patients and controls. Whole venous blood samples (10 mL) were collected in PaxGene Tubes at room temperature at the same time of the serum samples and then stored at −80 °C until they were processed. Total RNA was extracted from 1.5 mL of the whole blood using the Qiagen PAXGene Blood RNA Kit (QIAGEN GmbH, Hilden, Germany) according to the manufacturer’s protocols. Two micrograms of total RNA were used for cDNA synthesis and for subsequent gene expression analysis in real time PCR. Quantitative real-time PCR was performed using HOT FIREPol® EvaGreen® qPCR Mix (Solis BioDyne, Tartu, Estonia), according to the SYBR Green method. For each target primer set, a validation experiment was performed to demonstrate that PCR efficiencies were within the range of 90% to 100% and equal to the efficiencies of the reference genes (glyceraldehyde 3-phosphate dehydrogenase (GAPDH), beta-actin (ACTB), and beta-2- microglobulin (B2M)). Each sample was assayed in duplicate, and data presented regarding the target genes, IL-1α, IL-1β, IL-6, IL-8, and TNF-α, were normalized to the gene expression of the three reference genes: GAPDH, ACTB and B2M. Data were expressed as the relative expression ratio (R).

### 2.3. Statistical Analysis

The data were analyzed using Statistical software (SPSS V23, Inc., IL, USA). The normality of distribution for measurement data was tested using the Kolmogorov–Smirnov test. Count data are expressed as percentages or rates, and were compared using the chi-square test between groups; measurement data are presented as mean ± standard deviation and were compared using the *t*-test between the two groups for parametric data and the Mann–Whitney U test for non-parametric data, and analysis of variance (ANOVA) was used to compare different groups for parametric data and the Kruskal–Wallis test for non-parametric data. *P* < 0.05 indicates a significant difference. Serum cytokine levels were normalized for the statistical analyses through logarithmic transformation. Serum cytokines levels are presented as raw values, while the statistics were conducted on the logarithmic transformed values.

## 3. Results

Demographic and clinical characteristics of the participants are included in Table 1. A total of 640 participants were recruited for the current study including 440 epilepsy patients (240 males and 200 females; mean age ± SD: 29 ± 5.7 years) and 200 healthy controls (104 males and 96 females; mean age ± SD: 28.8 ± 5.3 years). Among the 440 studied epileptic patients, 165 (37.5%) demonstrated lifetime cannabis use (88 males and 77 females; mean age ± SD: 29.6 ± 5.8 years) with a mean duration of 15 ± 73 years. Two hundred and seventy-five epileptic patients were not using cannabis and were the so-called “Epilepsy-only” group (152 males and 123 females; mean age ± SD: 28.8 ± 5.9 years). Patients and controls did not significantly differ for age (F = 1.075, *df* = 2, *p*-value = 0.342) and sex (chi square = 0.514, *p*-value = 0.773). Differences between “Epilepsy-only” and “Epilepsy + Cannabis” groups were demonstrated in regards to the age of seizure onset, epilepsy duration, seizure type, type of therapy, seizure frequency, seizure severity, and adverse event profile (AEP). The percentage of patients demonstrating generalized and/or both seizure forms were significantly higher among the “Epilepsy-only” group (*p*-value = 0.05). No statistically significant differences were detected between the two patient groups in relation to the age of seizure onset (8.8 ± 1.7 vs. 8.5 ± 1.2, *p*-value = 0.117) and duration of seizures (20.3 ± 7.8 vs. 22.1 ± 7.7, *p*-value = 0.883). In regards to clinical epilepsy characteristics, the mean seizure frequency among the “Epilepsy-only” group was 45.7 ± 22.2 while in the “Epilepsy + Cannabis” group, it was 42.1 ± 12.2 (*p*-value = 0.656). Using the Chalfont seizures severity scale (CSSS) and the AEP revealed no significant differences between the two patient groups with *p*-value = 0.583 and 0.583, respectively. To reduce the effect of the known confounders, multiple analyses were conducted to test the differences of the cytokines’ concentrations in the two studied groups separately based on the pharmacological class of AEDs and in the whole epileptic patients as regards the gender, but no significant differences were encountered (Supplementary Tables S1–S4).

The majority of respondents (*n* = 165) reported that cannabis use had a positive impact on their epilepsy condition (67.9%, *n* = 112). The degree in which they agreed on the impact of cannabis on their illness varies from strongly agreed (10.3%, *n* = 17), agreed (57.6%, *n* = 95), neutral (22.4%, *n* = 37), disagree (6.1%, *n* = 10), and strongly disagree (3.6%, *n* = 6). One hundred and thirty-six (82.5%) patients stated that they did not know the type of the cannabis use, whether CBD or THC, and only a minority (17.5%, *n* = 29) answered that they use multiple types with different administration methods; smoking (69.7%, *n* =115), edibles (27.3%, *n* = 45), and drinks (7.8%, *n* = 13). Only 12 of 165 participants were able to give an exact dosage used in milligrams. Friends/family members and “dealers” were the most common cannabis sources. Although pharmaceutical CBD extract is now Food and Drug Administration (FDA)-approved for certain epilepsy types, access remains limited or even nonexistent in Egypt (Table 2).

Gene expression analyses conducted only in those cytokines that were different in the serum—IL-1α, IL-1β, IL-2, IL-6, IL-8, IL-10 and TNFα—revealed significantly different mRNA levels among both patient groups; “Epilepsy-only” and “Epilepsy + Cannabis” groups compared to healthy controls (*p*-value = 0.0001). When both epilepsy groups were further analyzed, the levels of mRNA showed significantly higher expression in the “Epilepsy-only” group compared to “Epilepsy + Cannabis” group for IL-1α (2.9 ± 1.4 vs. 2.1 ± 0.9, *p*-value = 0.0001), IL-1β (2.9 ± 1.4 vs. 2.1 ± 0.9, *p*-value = 0.0001), IL-2 (2.9 ± 1.4 vs. 2.1 ± 0.9, *p*-value = 0.0001), IL-6 (2.9 ± 1.4 vs. 2.1 ± 0.9, *p*-value = 0.0001), IL-8 (2.8 ± 1.4 vs. 2.1 ± 0.9, *p*-value = 0.0001), and TNF-α (2.8 ± 1.4 vs. 2.1 ± 0.9, *p*-value = 0.0001), while IL-10 was significantly lower in the “Epilepsy-only” group compared to the “Epilepsy + Cannabis” group (1.1 ± 0.5 vs. 1.4 ± 0.8, *p*-value = 0.002) (Table 4).

## 4. Discussion

In the first part of our study, the large number of patients surveyed reported successfully using cannabis outside of the medical system. Despite using varied combinations of THC:CBD as opposed to exclusively high CBD strains, the majority believe, with varying degree of agreement, that cannabis use helps them feel better about their epilepsy condition. One hundred and thirty-six out of 165 cannabis users replied that they did not know the exact content of the cannabis they use, and 29 out of the total number of cannabis users stated that they use multiple types that would probably encompass various ingredients with different purity. In the term of the amount used, patients were unable to quantify the exact amount (in milligrams) of cannabis they were using, which can partially be explained by the fact that the majority of them reported smoking as the primary means of administration which does not allow for precise dosage measurement. Instead, they replied as “indistinct measurement”, or sometimes, they describe it as “one dropper full” or “pea-sized amount”. However, the majority replied they use cannabis at least 1 to 2 times daily (34.5% for those answered once daily and 40.6% for the answers 1 to 2 times per day). They stated that it is finally a matter of availability and that the whole thing depends on socioeconomic reasons that would cause pure forms to be difficult to obtain. Patients surveyed did not have a full clear understanding of the potential risks and benefits of cannabis and its components. They used cannabis that contains varying amounts of THC. Although they stated that some pure preparations, especially “oil”, are available, the cost is still the main hindering factor limiting the use of CBD oil at dosages comparable to those used in clinical trials. Additionally, no commercial CBD oil or other preparations are available in Egypt and, rather, are prohibited.

Generally, epilepsy patients report that cannabis use is helpful for seizures as a way of justifying their recreational use or may be influenced by a strong desire for cannabis to be an effective treatment. In this context, in 2015, a retrospective study done in Colorado highlighted one aspect of self-reporting bias among patients taking medical cannabis for epilepsy treatment. Patients involved in the study moved to Colorado for the purpose of obtaining legal medical cannabis. They were 3 times more likely to report significant cannabis benefit than patients who were native to Colorado. This finding showed that the amount of effort that patients put forth to obtain medical cannabis affects their perception of treatment efficacy [51].

Regarding cannabis sources, it was logic to find out that “dealers” and friends/family members are the source because pharmacy and medical dispensaries are not legalized in Egypt to deal with any commercial or medical cannabis preparations. The majority of patients generally found cannabis more accessible nowadays than before. Gender differences in cannabis use might reflect environmental epidemiologic gender differences in recreational cannabis use, since more men than women use cannabis for recreational purposes in a lifetime sustained manner, and men are more likely than women to be diagnosed as having cannabis use disorder [52,53].

The above findings emphasize the knowledge gap among patients and doctors regarding the exact amount of cannabis and, ultimately, the appropriate dosing for medical usage in epilepsy disorder. Even when highly educated patients intend to use CBD oil similarly to the dosing used in clinical trials for epilepsy, they tend to use a sub therapeutic dose or not understand how much they are actually taking. This limitation in our study is in coherent with the fact that many cannabis dispensaries do mark products with respect to THC and CBD content [50]; however, multiple studies have demonstrated that the labeling can be inaccurate [54,55]. The number of doses per day varied among the participants in the study which is expected as cannabis was not being used as a medication; otherwise, one would expect it to be taken regularly in the same manner as other epilepsy medications. This hinders the evaluation of whether or not cannabis use has a real influence on the course of epilepsy disease. In this context we have encountered no significant differences between the two patient groups in the terms of seizure frequency and severity. Besides, there are many factors that may trigger the use of cannabis products on a different schedule than in the regular clinical trials, which may include cost, poor understanding or misunderstanding of cannabis effect, desire to use cannabis more frequently for recreational reasons, or conversely, desire to avoid using cannabis during the day to avoid feeling “high”.

The potential area where a patient can benefit from cannabis in the treatment of epilepsy is yet far in Egypt. Further studies and education may be beneficial in the future. The responses highlight the wide gap between patients’ desire to use cannabis products for recreational purposes and their knowledge about how to use cannabis as an epilepsy treatment. We propose that future research should be directed to assess the efficacy of physician counseling regarding the risks and benefits of medical cannabis. Indeed, clinical trials are mandatory to decide whether or not cannabidiol is an efficient treatment for epilepsy disease.

In the second part of the current work, we highlighted the link between epilepsy disease and the influence of cannabis use on inflammatory mediators as a putative mechanism of epilepsy. Although the role of inflammation in patients with epilepsy has been widely debated and partly considered as just an epiphenomenon, resulting from seizure-induced damage to the brain or excessive muscular activity, interestingly, the current findings of elevated levels of the inflammatory cytokines; IL-1α, IL-1β, IL-2, IL-6, IL-8, and TNF-α, both in serum and leukocytes gene expression analyses, is in agreement with the previously shown results of elevated cytokines levels in epilepsy patients [1,20,22,40,56,57,58] and moreover, provides direct clinical evidence that the immune system and cytokines mediators are involved in epilepsy disorder.

Generally, there is crosstalk between seizures and inflammatory mediators. Some epilepsy can originate from inflammatory changes or traumatic injuries early in life, ion channel malfunction, as well as metabolic or degenerative diseases [59,60,61]. The involvement of cytokines in the pathogenesis of epilepsy has been suggested by the evidence that limbic seizures increase mRNA of inflammatory cytokines in rodent forebrains [19]. It was also suggested that seizures trigger de novo synthesis of cytokines. An attractive hypothesis postulated that glutamate released from the neurons during seizures activates cytokine transcription in glial cells; however, specific effects triggering the seizures may play a role in this activation such as protein extravasations from the BBB or stress associated events priming glial cells [62,63,64,65]. In the same context, it was demonstrated that both humoral and cell-mediated immunity are more functionally disturbed among persons with the epilepsy than in the general population [66,67,68,69].

The serum and gene expression of the inflammatory cytokines—IL-1α, IL-1β, IL-2, IL-6, IL-8 and TNF-α—showed the highest levels among the “Epilepsy-only” group followed by lower levels in the epilepsy–cannabis users and finally the lowest levels in the healthy controls. Our findings of the lower inflammatory cytokine levels among cannabis users in comparison to the epileptic non-cannabis users are not surprising, and are coherent with previous pre-clinical and clinical studies showing an anti-inflammatory effect of cannabinoids [70,71,72,73]. Interestingly, the anti-inflammatory IL-10 was significantly lower in both epilepsy groups compared to healthy controls; however, it showed a significant higher level in the “Epilepsy + Cannabis” group when compared to the “Epilepsy-only group”. IL-10 possesses an essential regulatory, anti-inflammatory role but the mechanisms that lead to IL-10 production during infection and inflammation are incompletely understood [1]. IL-10 opposes many pro-inflammatory processes during diseases. It may ameliorate immunopathology by preventing inflammation-associated tissue damage [74]. Our finding of increased levels of IL-10 among epileptic cannabis users compared to epileptic-only patients might explain partly the anti-inflammatory effect of cannabis that would serve as a clue for its anticonvulsant effect. However, some studies have demonstrated elevated levels of IL-10 among epileptic patients similar to the pro-inflammatory cytokines [1]. The increased IL-10 in epilepsy patients can be due to counteracting mechanisms to the pro-inflammatory stimuli.

In contrary, IL-4 showed no significant difference between patients and controls nor when the comparison has been drawn in regards to cannabis use. The earliest studies of IL-4 in macrophages showed that it represents an anti-inflammatory agent when administered concurrently or shortly after an inflammatory stimulus, and is capable of downregulating the production of inflammatory cytokines such as TNF [75]. Moreover, IL-4 is not only purely an anti-inflammatory agent, however, as the priming of macrophages with IL-4 followed by pro-inflammatory stimulation can yield an enhanced inflammatory response [76]. In vivo studies have revealed that chronic high dosage or transgenic overproduction of IL-4 results in accumulation of AAMΦs, increased IFN-γ expression, decreased pro-inflammatory cytokine production, histiocytosis, erythrophagocytosis, extramedullary hematopoiesis, and weight loss [77]. Accordingly, it was suggested that the in vivo effects of IL-4 are complex, well regulated, dependent on the local environment, and that they probably intermediate different processes simultaneously in different tissues. The situation is even more complex in the context of the brain, which is clearly affected by IL-4; however, little is known about the ability of this cytokine to access the parenchyma or the nature of its effects on target cells. IL-4 has been studied in microglia and astrocytes within the CNS; nevertheless, the possible role of IL-4 in directly stimulating neurons and oligodendrocytes is poorly understood [78].

Discrepancies of our findings to other studies might result from the post-transcriptional abnormalities of cytokine expression, leading to a lack of correlation between transcript levels and protein levels. Gene expression may be more easily and rapidly affected than systemic cytokine levels [79], and therefore some differences between gene expression and serum levels might be related to the effects of more recent and acute vs. chronic activation of the immune system. Indeed, elevated cytokines may derive from cytokine-producing cells other than peripheral immune cells, such as intra-hepatic or adipose tissue-associated cells [80].

## 5. Conclusions

In conclusion, the present data indicate that inflammatory cytokines correlated with seizure and that both cytokines serum and gene expression levels are elevated among epilepsy patients more than the normal population. Cannabis use is prevalent among epilepsy patients although neither recreationally nor medically legalized. Despite the absence of clinical evidence of seizure improvement in regards to severity and frequency, our primary evidences showed that cannabis use influence cytokines serum levels and gene expressions resulting in less inflammatory and higher anti-inflammatory flow. This might support the concept behind pre-clinical and clinical trials using cannabidiol as an anticonvulsant drug for treatment-resistant epilepsy. Further well-designed studies and RCTs are needed in the future to answer whether or not cannabis as a treatment for epilepsy fulfills its promise.

## Figures and Tables

**Table 1:** Demographic data and clinical characteristics of the study participants (*n* = 640).

	Control	Epilepsy-Only	Epilepsy and Cannabis	Combined ^a^	*p*-Value
Number of cases	200	275	165	440	
Sex Male Female	104 (52%) 96 (48%)	152 (55.3%) 123 (44.7%)	88 (53.3%) 77 (46.7%)	240 (54.5%) 200 (45.5%)	0.773 ^c^
Age at enrollment (years)	28.8 ± 5.3	28.8 ± 5.9	29.6 ± 5.8	29 ± 5.7	0.342 ^c^
Duration of cannabis use			15 ± 73		
Age at seizure onset (years)		8.8 ± 1.7	8.5 ± 1.2	8.7 ± 1.5	0.117 ^d^
Epilepsy duration (years)		20.3 ± 7.8	22.1 ± 7.7	20.9 ± 7.8	0.883 ^d^
Seizure type Partial Generalized Both		231 (84%) 23 (8.4%) 21 (76%)	152 (92.1%) 7 (4.2%) 6 (3.6%)	383 (87%) 30 (6.8%) 27 (6.1%)	0.05 ^d^
Type of therapy Monotherapy Polytherapy		66 (24%) 209 (76%)	53 (32.1%) 112 (67.9%)	119 (27%) 321 (73%)	0.063 ^d^
Mean seizure frequency at enrollment		45.7 ± 22.2	42.1 ± 12.2	44.4 ± 20.8	0.657 ^d^
Seizure severity at enrollment		83.7 ± 49.1	78.9 ± 62.8	80.7 ± 56.6	0.583 ^d^
AEP ^b^ at enrollment		42.1 ± 10.1	39.6 ± 9.1	40.4 ± 9.5	0.583 ^d^

**^a^** Combined = Epilepsy-only and Epilepsy + Cannabis, **^b^** Adverse event profile, **^c^** Comparison between control group and the studied groups in reference to “age” using one way ANOVA to compare means and sex using a chi-square test, **^d^** Comparison between Epilepsy-only and Epilepsy + Cannabis groups in reference to epilepsy criteria using a chi-square for categorical variables (qualitative data), a *t*-test for comparison of parametric quantitative data and the Mann–Whitney U-test for comparison of non-parametric quantitative data.

**Table 2:** Summary of cannabis use survey responses (*n* = 165).

Question	Answer Option	Responses (*n* = 165)	Percent
Sex	Male Female	88 77	53.3% 46.7%
How long do you use cannabis?	Years	15±73	
Do you think that cannabis improves your epilepsy? Or do you use cannabis to treat your seizures?	Yes No	112 53	67.9 32.1
How do you use cannabis? Check as many as apply	Smoking Vaping Bong/waterpipes Edibles Topical Drinks (tea, soda) Tinctures Other concentrates	115 3 2 45 5 13 0 0	69.7 1.8 1.2 27.3 3.03 7.8 0 0
How can you obtain cannabis? From where you buy?	Home grown Family/friends/job colleagues Recreational shops/dealers Pharmacy Medical dispensary	0 165 165 0 0	0 100 100 0 0
Do you think getting cannabis has become easier or harder in the latest years?	Easier to get Harder to get Same as before	150 3 12	90.9 1.8 7.3
In a typical week, how many times do you use cannabis?	Less than once a week 1-2 times 3-6 times Once daily Several times daily I do not know	0 67 18 57 13 10	0 40.6 10.9 34.5 7.9 6.1
How many milligrams do you use each time?	I don’t know or indistinct measurement 0.5–10 mg 10–100 mg 100–1000 mg >1 g (flower weight)	153 3 3 5 1	92.7 1.8 1.8 3.1 0.6
Do you the content of your cannabis?	More CBD More THC Equal CBD and THC I use multiple types I don’t know	0 0 0 29 136	0 0 0 17.5 82.5
To what extent do you agree with the following sentence: “cannabis improved my epilepsy condition”	Strongly agree Agree Neither agree nor disagree (neutral) Disagree Strongly disagree	17 95 37 10 6	10.3 57.6 22.4 6.1 3.6

Serum levels of IL-1α, IL-1β, IL-2, IL-6, IL-8, and TNF-α were significantly higher among both patient groups; “Epilepsy-only” and “Epilepsy + Cannabis” groups compared to healthy controls (*p*-value = 0.0001). Furthermore, patients using cannabis had significantly lower levels of inflammatory cytokines when compared with patients who did not use cannabis; IL-1α (12.3 ± 5.7 vs. 21.2 ± 8.8, *p*-value = 0.0001), IL-1β (28.7 ± 9.3 vs 31.5 ± 12.1, *p*-value = 0.048), IL-2 (17.7 ± 6.8 vs. 40.1 ± 10.2, *p*-value = 0.0001), IL-6 (17.8 ± 6.7 vs. 43.5 ± 12.8, *p*-value = 0.0001), IL-8 (21.3 ± 7.6 vs. 43.5 ± 12.8, *p*-value = 0.0001), and TNF-α (71.9 ± 16.1 vs. 123.3 ± 32.1, *p*-value = 0.0001), respectively. In contrary, mean levels of IL-10 among “Epilepsy-only” and “Epilepsy + Cannabis” groups were significantly lower than the control healthy subjects (13.9 ± 4.5, 24.6 ± 6.9, 43.7 ± 12.9, respectively; *p*-value = 0.0001); however, the IL-10 serum level was significantly higher among the “Epilepsy + Cannabis” group in comparison to “Epilepsy-only” (24.6 ± 6.9 vs. 13.9 ± 4.5; *p*-value = 0.0001). No significant differences were detected when the mean serum level of IL-4 were compared between groups (“Epilepsy-only” vs. “Control” group *p*-value = 0.841, “Cannabis+Epilepsy” vs “Control” *p*-value = 0.097, and “Epilepsy-only” vs. “Cannabis+Epilepsy” *p*-value = 0.898) (Table 3).

**Table 3:** Comparisons of the serum inflammatory cytokine levels among the Control group, the Epilepsy-only group and the Epilepsy + Cannabis group.

Inflammatory Cytokines	Control Group Mean ± SD	Epilepsy-Only Mean ± SD	Cannabis+Epilepsy Mean ± SD	*p*-value ^a^
IL-1α (pg/mL)	4.6 ± 2.9	21.2 ± 8.8	12.3 ± 5.7	0.0001
	Epilepsy-only vs. Control group 0.0001	Cannabis+Epilepsy vs. Control 0.0001	Epilepsy-only vs. Cannabis+Epilepsy 0.0001	
IL-1β (pg/mL)	3.7 ± 2.9	31.5 ± 12.1	28.7 ± 9.3	0.0001
	Epilepsy-only vs. Control group 0.0001	Cannabis+Epilepsy vs. Control 0.0001	Epilepsy-only vs. Cannabis+Epilepsy 0.048	
IL-2 (pg/mL)	5.9 ± 1.7	40.1 ± 10.2	17.7 ± 6.8	0.0001
	Epilepsy-only vs. Control group 0.0001	Cannabis+Epilepsy vs. Control 0.0001	Epilepsy-only vs. Cannabis+Epilepsy 0.0001	
IL-4 (pg/mL)	14.6 ± 5.1	13.8 ± 4.9	13.7 ± 5.1	0.150
	Epilepsy-only vs. Control group 0.841	Cannabis+Epilepsy vs. Control 0.097	Epilepsy-only vs. Cannabis+Epilepsy 0.898	
IL-6 (pg/mL)	5.8 ± 1.8	43.5 ± 12.8	17.8 ± 6.7	0.0001
	Epilepsy-only vs. Control group 0.0001	Cannabis+Epilepsy vs. Control 0.0001	Epilepsy-only vs. Cannabis+Epilepsy 0.0001	
IL-8 (pg/mL)	23.8 ± 6.2	43.5 ± 12.8	21.3 ± 7.6	0.0001
	Epilepsy-only vs. Control group 0.0001	Cannabis+Epilepsy vs. Control 0.0001	Epilepsy-only vs. Cannabis+Epilepsy 0.0001	
IL-10 (pg/mL)	43.7 ± 12.9	13.9 ± 4.5	24.6 ± 6.9	0.0001
	Epilepsy-only vs. Control group 0.0001	Cannabis+Epilepsy vs. Control 0.0001	Epilepsy-only vs. Cannabis+Epilepsy 0.0001	
TNF-α (pg/mL)	36.1 ± 6.9	123.3 ± 32.1	71.9 ± 16.1	0.0001
	Epilepsy-only vs. Control group 0.0001	Cannabis+Epilepsy vs. Control 0.0001	Epilepsy-only vs. Cannabis+Epilepsy 0.0001	

^**a**^ The comparison between the three presented groups using Kruskal–Wallis and Mann–Whitney U-tests for analysis of non-parametric data. *P*-value < 0.05 is considered significant. IL-1α = interleukin 1 alpha; IL-1β = interleukin 1 beta; IL-2 = interleukin 2; IL-4 = interleukin 4; IL-6 = interleukin 6; IL-8 = interleukin 8; IL-10 = interleukin 10; TNF-α = tumor necrosis factor alpha.

**Table 4:** Comparisons of the cytokine gene expression among the Control group, the Epilepsy-only group and the Epilepsy + Cannabis group.

Inflammatory Cytokines	Control Group Mean ± SD	Epilepsy-Only Mean ± SD	Cannabis+Epilepsy Mean ± SD	*p*-value ^a^
IL-1α mRNA (R)	1.1 ± 0.6	2.9 ± 1.4	2.1 ± 0.9	0.0001
	Epilepsy-only vs. Control group 0.0001	Cannabis+Epilepsy vs. Control 0.0001	Epilepsy-only vs. Cannabis+Epilepsy 0.0001	
IL-1β mRNA (R)	1.1 ±0.6	2.9 ± 1.4	2.1 ± 0.9	0.0001
	Epilepsy-only vs. Control group 0.0001	Cannabis+Epilepsy vs. Control 0.0001	Epilepsy-only vs. Cannabis+Epilepsy 0.0001	
IL-2 mRNA (R)	1.1 ± 0.6	2.9 ± 1.4	2.1 ± 0.9	0.0001
	Epilepsy-only vs. Control group 0.0001	Cannabis+Epilepsy vs. Control 0.0001	Epilepsy-only vs. Cannabis+Epilepsy 0.0001	
IL-6 mRNA (R)	1.1 ± 0.6	2.9 ± 1.4	2.1 ± 0.9	0.0001
	Epilepsy-only vs. Control group 0.0001	Cannabis+Epilepsy vs. Control 0.0001	Epilepsy-only vs. Cannabis+Epilepsy 0.0001	
IL-8 mRNA (R)	1.1 ± 0.5	2.8 ± 1.4	2.1 ± 0.9	0.0001
	Epilepsy-only vs. Control group 0.0001	Cannabis+Epilepsy vs. Control 0.0001	Epilepsy-only vs. Cannabis+Epilepsy 0.0001	
IL-10 mRNA (R)	2.8 ± 1.3	1.1 ± 0.5	1.4 ± 0.8	0.0001
	Epilepsy-only vs. Control group 0.0001	Cannabis+Epilepsy vs. Control 0.0001	Epilepsy-only vs. Cannabis+Epilepsy 0.002	
TNF-α mRNA (R)	1.1 ± 0.5	2.8 ± 1.4	2.1 ± 0.9	0.0001
	Epilepsy-only vs. Control group 0.0001	Cannabis+Epilepsy vs. Control 0.0001	Epilepsy-only vs. Cannabis+Epilepsy 0.0001	

^**a**^ The comparison between the three presented groups using Kruskal–Wallis and Mann–Whitney U-tests for analysis of non-parametric data. *p*-value < 0.05 is considered significant. mRNA = messenger ribonucleic acid; IL-1α = interleukin 1 alpha; IL-1β = interleukin 1 beta; IL-2 = interleukin 2; IL-4 = interleukin 4; IL-6 = interleukin 6; IL-8 = interleukin 8; IL-10 = interleukin 10; TNF-α = tumor necrosis factor alpha.

## Supplementary Materials

The following are available online at https://www.mdpi.com/2076-3425/9/12/332/s1, Table S1: Comparison of the mean serum cytokine levels and mRNA gene expression profile among patients on monotherapy (using Na-valproate) (*n* = 119) as regard cannabis use, Table S2: Comparison of the mean serum cytokine levels and mRNA gene expression profile among epileptic patients using cannabis “Epilepsy + Cannabis” (*n* = 165) as regard AEDs, Table S3: Comparison of the mean serum cytokine levels and mRNA gene expression profile among epileptic patients (*n* = 440) as regard sex, Table S4: Comparison of the mean serum cytokine levels and mRNA gene expression profile among epileptic patients using cannabis and on monotherapy (*n* = 53) as regard sex.
