# Supplementary material for: Cannabis Influences the Putative Cytokines-Related Pathway of Epilepsy among Egyptian Epileptic Patients
Source: Brain Sci. 2019 Nov 20;9(12):332. doi: 10.3390/brainsci9120332 (PMC6955862; doi:10.3390/brainsci9120332)
Supplement: Supplementary file 1 [file brainsci-09-00332-s001.pdf]

Supplementary Tables

In the current study, three variables could possibly affect the cytokines levels; sex, type of therapy and/or cannabis. Therefore, we conducted three comparisons to avoid the potential confounders:

First comparison of the cytokines' levels among the "epileptic patients on monotherapy/ Na-valproate" (n=119) as regard cannabis use which as confounding factor was distributed unequally among those patients. This comparison was used to avoid the confounding effect of the AEDs. This resulted in statistically significant differences in all inflammatory cytokines except IL-1β, IL-104 and IL-10. This denotes that cannabis display real effect on the inflammatory cytokines among epileptic patients.

Table S1: Comparison of the mean serum cytokine levels and mRNA gene expression profile among patients on monotherapy (using Na-valproate) (n=119) as regard cannabis use.

Serum and mRNA Cytokines levels	Epilepsy+Cannabis (n=53)	Epilepsy only (Non-cannabis)(n=66)	***p***-value
IL-1α (pg/ml)	10.8±5.5	22.9±9.2	0.003
IL-1β (pg/ml)	28.2± 9.7	29.6± 12.1	0.19
IL-2 (pg/ml)	17.2± 6.9	40.8± 11.9	0.0001
IL-4 (pg/ml)	13.7± 5.3	14.1± 5.4	0.571
IL-6 (pg/ml)	17.2± 6.9	42.9± 13.2	0.0001
IL-8(pg/ml)	21.3± 8.7	44.6± 14.4	0.0001
IL-10 (pg/ml)	23.6± 6.2	14.3± 4.8	0.297
TNFα(pg/ml)	68.8± 15.9	128.9± 29	0.005
IL-1α mRNA (R)	2.2±1.1	2.6±1.3	0.02
IL-1β mRNA (R)	2.2±0.9	2.7±1.3	0.007
IL-2 mRNA (R)	2.4±1.1	2.7±1.3	0.03
IL-6 mRNA (R)	2.2±1.1	2.6±1.3	0.02
IL-8 mRNA (R)	2.1± 0.8	3.1± 1.3	0.001
IL-10 mRNA (R)	1.4± 0.9	1.1± 0.3	0.001
TNFα mRNA (R)	2.1± 0.8	3.1± 1.3	0.001

Second comparison was to test the effect of AEDs in our studied population sample and in this case, to avoid the confounding effect of cannabis. We analyzed the differences in the cytokines' levels between monotherapy and polytherapy among "Epilepsy+Cannabis" however, no significant differences were detected.

Table S2: Comparison of the mean serum cytokine levels and mRNA gene expression profile among epileptic patients using cannabis "Epilepsy+Cannabis" (n=165) as regard AEDs.

Serum and mRNA Cytokines levels	Monotherapy (n=53)	Polytherapy (n=112)	***p***-value
IL-1α (pg/ml)	10.8± 5.5	12.9± 5.7	0.933
IL-1β (pg/ml)	28.2± 9.7	28.9± 9.2	0.531
IL-2 (pg/ml)	17.2± 6.9	17.9± 6.7	0.944
IL-4 (pg/ml)	13.7± 5.3	13.7± 4.9	0.951
IL-6 (pg/ml)	17.2± 6.9	18.1± 6.7	0.904
IL-8(pg/ml)	21.3± 8.7	21.4± 7.1	0.113
IL-10 (pg/ml)	23.6± 6.2	25.1± 7.3	0.128
TNFα(pg/ml)	68.8± 15.9	73.4± 15.1	0.436
IL-1α mRNA (R)	2.2± 1.1	2.1± 0.9	0.728
IL-1β mRNA (R)	2.2± 0.9	2.1± 0.9	0.654
IL-2 mRNA (R)	2.2± 1.1	2.1± 1	0.599
IL-6 mRNA (R)	2.2± 1	2.1± 0.9	0.708
IL-8 mRNA (R)	2.1± 0.8	2.1± 0.9	0.174
IL-10 mRNA (R)	1.4± 0.9	1.4± 0.8	0.653
TNFα mRNA (R)	2.1± 0.8	2.1± 0.9	0.185

Third comparison was to avoid the confounding effect of sex. We analyzed the differences in the cytokines' levels once among the whole studied epileptic patients and then among epileptic patients using cannabis and on monotherapy.

Table S3: Comparison of the mean serum cytokine levels and mRNA gene expression profile among epileptic patients (n=440) as regard sex.

	Male	Females	p-value
IL-1α (pg/ml)	14.1±9.6	13.3±9.8	0.743
IL-1β (pg/ml)	21.8±15.3	22.3±15.9	0.376
IL-2 (pg/ml)	24.2±16.9	23±16.6	0.431
IL-4 (pg/ml)	14.1±4.9	14.1±5.1	0.647
IL-6 (pg/ml)	25.2±18.4	24.8±19.5	0.454
IL-8 (pg/ml)	32.5±15.1	32.3±15.2	0.832
IL-10 (pg/ml)	25.4±15.3	26.6±15.4	0.939
TNF- α (pg/ml)	83.2±43.3	82.3±45.1	0.270
IL-1α mRNA (R)	2.2±1.3	2.1±1.4	0.611
IL-1β mRNA (R)	2.2±1.3	2.1±1.4	0.467
IL-2 mRNA (R)	2.2±1.3	2.1±1.4	0.528
IL-6 mRNA (R)	2.2±1.4	2.1±1.4	0.691
IL-8 mRNA (R)	2.1±1.1	2.1±1.3	0.211
IL-10 mRNA (R)	1.7±1.1	1.7±1.2	0.314
TNF- α mRNA (R)	2.1±1.2	2.1±1.2	0.305

Table S4: Comparison of the mean serum cytokine levels and mRNA gene expression profile among epileptic patients using cannabis and on monotherapy (n=53) as regard sex.

	Male	Females	p-value
IL-1α (pg/ml)	11.6± 4.8	10.1± 6.2	0.071
IL-1β (pg/ml)	28.1± 10.2	28.4± 9.3	0.734
IL-2 (pg/ml)	16.8± 6.1	17.6± 7.6	0.114
IL-4 (pg/ml)	15.1± 5.2	12.1± 5.2	0.394
IL-6 (pg/ml)	16.8± 6.1	17.6± 7.6	0.114
IL-8 (pg/ml)	20.5± 8.5	22.1± 8.9	0.431
IL-10 (pg/ml)	23.9± 7.1	23.2± 5.2	0.354
TNF- α (pg/ml)	70.9± 14.2	66.7± 17.5	0.292
IL-1α mRNA (R)	2.3± 0.9	2.1± 1.1	0.556
IL-1β mRNA (R)	2.3± 0.9	2.1± 1.1	0.556
IL-2 mRNA (R)	2.3± 0.9	2.1± 1.1	0.648
IL-6 mRNA (R)	2.3± 0.9	2.1± 1.1	0.556
IL-8 mRNA (R)	2.1± 0.7	2.1± 0.9	0.167
IL-10 mRNA (R)	1.4± 0.8	1.4± 0.9	0.579
TNF- α mRNA (R)	1.9± 0.7	2.1± 0.9	0.132
